# Shifts in clinical practice and patient demographics following the introduction of holmium laser enucleation for benign prostatic hyperplasia in a general urology clinic

**DOI:** 10.14440/jbm.2025.0002

**Published:** 2025-08-20

**Authors:** Ankur U. Choksi, Shayan Smani, Soum D. Lokeshwar, Vinaik M. Sundaresan, Christopher S. Hayden, Daniel A. Segal, Daniel S. Kellner

**Affiliations:** Department of Urology, Yale School of Medicine, Yale University, New Haven, Connecticut 06510, United States of America

**Keywords:** Benign prostatic hyperplasia, Holmium laser enucleation of the prostate, Ambulatory, Prostate surgery

## Abstract

**Background:**

Holmium laser enucleation of the prostate (HoLEP) has emerged as an effective surgical treatment for benign prostatic hyperplasia (BPH). This study evaluated how the adoption of HoLEP in a general urology clinic influenced clinical and procedural volume.

**Objective:**

To better understand the practice ramifications of HoLEP adoption, we analyzed the changes to a general urologist’s patient demographics and practice patterns after the addition of HoLEP to their surgical repertoire.

**Methods:**

We retrospectively reviewed the electronic health records 30 months before and after the introduction of HoLEP to examine changes in a general urologist’s patient population. Pearson’s Chi-squared test and Student’s *t*-test were used for statistical analysis.

**Results:**

A total of 4390 unique patients were seen over a period of 5-years, with 2052 seen before and 2338 after the introduction of HoLEP. The mean distance from patients’ residence zip codes to the treatment center remained statistically unchanged (pre-HoLEP: 32.52 ± 152.42 miles, post-HoLEP: 29.65 ± 141.79 miles, *p*=0.9896). Among those who underwent HoLEP, prostate sizes were comparable between patients residing in the same county and those coming from different counties (96.42 ± 3.24 cc vs. 104.52 ± 4.34 cc, *p*=0.141). Surgical volume rose from 355 to 1018 cases with a concordant increase in other BPH-related surgeries, marked by an inflection point at the time of HoLEP’s introduction.

**Conclusion:**

There was an increase in clinical and surgical volume to an established general urologist’s practice after HoLEP was offered. Most patients continued to be drawn from the initial catchment area, potentially reflecting previously unmet treatment needs for patients with large prostate glands.

## 1. Introduction

The aging population of the United States is driving a rapid rise in the prevalence of benign prostatic hyperplasia (BPH), a commonly managed urological condition.^1^ Lower urinary tract symptoms are a constellation of manifesting symptoms of BPH, affecting 24% of men older than 70.^2^,^3^ For medication-refractory lower urinary tract symptoms, the gold standard for endoscopic prostatic adenoma resection has been the transurethral resection of the prostate (TURP).^4^ In the past two decades, new surgical modalities and improvements in technology have reduced the morbidity rate and cost of endoscopic management of BPH. Recently, holmium laser enucleation of the prostate (HoLEP) has emerged as a treatment alternative to TURP. HoLEP has been recognized for its long-term durability, shorter hospital stays, and low complication rate for men with moderate- and large-sized prostates.^5^-^8^ The European Association of Urology recommends HoLEP as the first-line surgical treatment for BPH in prostates larger than 80 g.^9^ In addition, the American Urological Association recognizes laser enucleation of the prostate as the only size-independent BPH surgical modality.^10^ As such, there is mounting evidence to support general urologists incorporating laser enucleation methods such as HoLEP into their practice.

Despite its benefits, HoLEP has not gained significant popularity in the United States, accounting for only 4% of BPH surgeries in 2014.^11^ Significant barriers hinder its wider adoption. The steep learning curve associated with HoLEP proficiency has been a significant barrier to its widespread use. A systematic review estimated the learning curve to range between 20 and 60 cases, while several studies reported even longer learning periods.^12^,^13^ Furthermore, HoLEP requires additional specialized equipment, such as a dedicated laser resectoscope sheath and morcellator, increasing the expense for the treatment.^14^ Despite the high upfront cost and technical challenge, Medicare/Medicaid reimbursement of HoLEP is roughly equivalent to that of TURP. Partly due to these factors, HoLEP training is not nearly as ubiquitous as its benefits would suggest. For individuals who are considering the incorporation of HoLEP into their surgical repertoire, it is important to understand the anticipated impact on their practice. To that end, we analyzed the changes to a self-trained general urologist’s patient demographics and practice patterns after the addition of HoLEP to their surgical repertoire. We hypothesized that the adoption of HoLEP would lead to a more extensive geographic distribution of referrals and directly lower the number of TURPs performed by the surgeon.

## 2. Methodology

We performed a retrospective analysis on a single general urologist’s patient population before and after offering HoLEP. Using the electronic health record, we analyzed office and surgical visits 30 months before and after the introduction of HoLEP to the surgeon’s practice. All surgeries were performed by a single, self-taught surgeon who gained experience in HoLEP through patient cases, supplemented by workshops and simulation training. Patient populations were grouped into patients treated before and after the introduction of HoLEP. Patient eligibility for HoLEP versus TURP was determined by a combination of surgeon and patient preferences, as well as prostate size. We assessed patient-specific demographics in terms of age, gender, and zip codes of primary residence. We also examined differences in primary diagnosis for office visits and surgical procedures performed by the single surgeon before and after adopting HoLEP. Outcomes of interest included changes in clinical and surgical volume, proportion of BPH-related surgeries, and changes in patient demographics. Patients’ resident zip codes were summarized using a geographic heatmap to assess for changes before and after the introduction of HoLEP. Finally, for patients who underwent HoLEP, prostate size was recorded as measured on magnetic resonance imaging.

Categorical variables were reported as frequencies and proportions (*n* [%]). Statistical analysis was conducted to compare patient and clinical populations before and after the introduction of HoLEP. A choropleth map was created to understand geographical variations in patient populations seeking medical care before and after the adoption of HoLEP. Categorical variables were analyzed using the Pearson’s Chi-squared test of independence. For each test result, a corresponding two-tailed *p*<0.05 was considered statistically significant. All analyses were performed using the Statistical Package for the Social Sciences v.28.0 software (IBM Corp., United States of America).

## 3. Results

**Figure 1:**
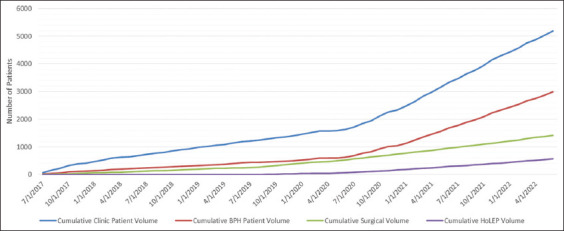
Cumulative frequency of clinical volume of clinic patients and surgical volume with respect to holmium laser enucleation of the prostate adoption Abbreviations: BPH: Benign prostatic hyperplasia; HoLEP: Holmium laser enucleation of the prostate.

A total of 4390 clinic visits with unique patients occurred over the 5-year period, with 2052 (46.7%) patient visits taking place prior to the introduction of HoLEP and 2338 (53.3%) unique patient visits being made thereafter. Table 1 displays a summary of patient demographics and surgical case volume. There were significantly more patients seen in the clinic for BPH after HoLEP’s introduction, with an increment from 893 (43.5%) to 1555 (66.5%) patients (Figure 1). The proportion of male patients seen in the clinic also increased after the practice changed, from 90.4% to 96.3%. Surgical volume rose from 355 to 1018 cases, alongside an increase in other BPH-related surgeries, from 77 before the introduction of HoLEP to 623 afterward.

**Table 1:** Clinic and patient demographics before and after the introduction of holmium laser enucleation of the prostate

Parameter	Frequency	Frequency	Significance
Total number of clinic patients	2052	2338	<0.01
Number of clinic patients with benign prostatic hyperplasia	893 (43.5)	1555 (66.5)	<0.01
Number of male patients	1856 (90.4)	2252 (96.3)	<0.01
Age of clinic patients			<0.01
<49 years	343 (16.7)	131 (6.8)	-
50–64 years	504 (24.5)	494 (25.6)	-
65–74 years	615 (30.0)	866 (44.9)	-
75–84 years	415 (20.2)	232 (12.0)	-
85+years	166 (8.1)	204 (10.6)	-

Note: Data are presented as *n* (%).

The most performed procedure before the introduction of HoLEP was ureteroscopy with laser lithotripsy and stent placement, followed by robotic radical prostatectomies and TURPs. While the surgeon performed more TURPs after the introduction of HoLEP, TURPs accounted for a smaller percentage of their total case volume. In addition, the number of robotic prostatectomies performed dropped significantly, with 64 cases performed prior to the introduction of HoLEP and 20 cases performed after (Table 2).

**Table 2:** Operations performed before and after the introduction of holmium laser enucleation of the prostate

Operation	Frequency	Frequency	Significance
Transurethral resection of the prostate	63 (17.7)	87 (8.5)	-
Transurethral incision of the prostate	14 (3.9)	20 (1.9)	-
Enucleation of the prostate	0 (0.0)	516 (50.7)	-
Ureteroscopy/laser lithotripsy/stent	130 (36.6)	164 (16.1)	-
Robotic radical prostatectomy	64 (18.0)	20 (1.9)	-
Other	84 (23.7)	211 (20.7)	-
Total	355	1018	<0.01

Note: Data are presented as *n* (%).

**Figure 2:**
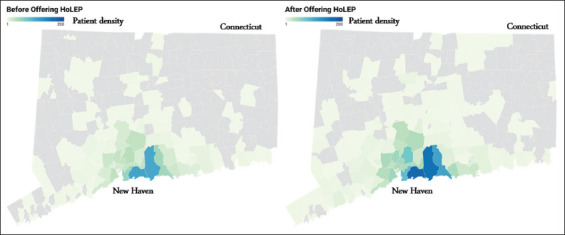
Geographic distribution of the zip code of primary residence for patients evaluated for BPH, before and after introduction of HoLEP. Image created with Datawrapper Abbreviations: BPH: Benign prostatic hyperplasia; HoLEP: Holmium laser enucleation of the prostate.

Figure 2 demonstrates the geographic distribution of the zip code of primary residence for patients being referred to the urologist for BPH, before and after HoLEP’s introduction. Most patients who underwent HoLEP lived within the same county as the treatment center. The geographic distribution of patients following HoLEP’s introduction remained unchanged. In addition, the mean distance of zip code of residence for patients with BPH to the treatment center also stayed statistically unchanged after including HoLEP in the treatment repertoire (pre-HoLEP: 32.52 ± 152.42 miles, interquartile range: 6.01–16.91; post-HoLEP: 29.65 ± 141.79 miles, interquartile range: 6.01–16.91; *p*=0.9896). Average prostate volumes were not significantly different based on the patients’ county of referral within the same county (96.42 ± 3.24 cc vs. 104.52 ± 4.34 cc, *p*=0.141). Similarly, there was no difference either in average prostate volume between patients referred from within the state and those referred from out of state (99.24 ± 2.61 cc vs. 117.48 ± 11.11 cc, *p*=0.069).

## 4. Discussion

Our retrospective analysis demonstrated a significant increase in clinical and surgical volume in a general urologist’s practice after the introduction of HoLEP. Although we hypothesized a change in the geographic distribution of patients after the introduction of HoLEP, this was not observed. Most patients continued to be referred from the same county as the treatment center, suggesting potential under-treatment of larger prostate glands that may have been deemed endoscopically unresectable by TURP. While the volume of other BPH-related treatments also rose—likely being related to the overall increase in BPH-related referrals—the number of robotic radical prostatectomies decreased, reflecting a practice shift from oncological procedures toward BPH-related procedures. This shift toward BPH-related procedures is likely common among practices adopting HoLEP in regions with few HoLEP-trained urologists.

While HoLEP is considered a size-independent approach to BPH surgery, current American Urological Association guidelines have recommended that several factors be considered, such as patient prostate size, the desire to preserve ejaculatory function, bleeding risk, and the presence of an obstructive median lobe, to tailor surgical recommendations, rather than uniformly offering a single procedure.^10^ For this surgeon, the clinical case volume expanded virtually threefold after the introduction of HoLEP. While a majority of the cases were HoLEP, the volume of other BPH-related surgeries, including TURPs, also increased following its introduction. During the 30 months after the introduction of HoLEP, the number of clinic referrals for BPH went up from 893 to 1555 unique patients, which likely explains the concurrent rise in other BPH treatments. Due to the increase in BPH referrals, other urological conditions, such as prostate cancer, may need to be treated by other individuals within the practice, as demonstrated by a decrease in the number of robotic prostatectomies performed. Prior studies have demonstrated that the adoption of HoLEP resulted in an increase in patients seeking HoLEP.^15^ These patients included those with larger prostate glands or prostates with large intravesical median lobes that were not amenable to TURPs. Our findings demonstrated that being able to surgically perform HoLEP increases the volume of patients seeking definitive urological care. Prior studies have also demonstrated no reduction in the overall number of TURPs performed after the introduction of HoLEP. The findings align with our results, which demonstrated an increase in TURP procedures performed following the adoption of HoLEP.^15^ In addition, with appropriate patient selection between HoLEP and TURP, outcomes for each procedure are likely to improve, as patients are better matched to the most suitable surgical option.^16^-^18^

Contrary to our hypothesis, the geographic distribution of patient referrals did not change significantly after adopting HoLEP. This may be attributed to the under-treatment of larger prostate glands within the surgeon’s existing catchment area and referral networks—patients previously considered unsuitable for TURP due to gland size or medical comorbidities being managed non-operatively. However, given the increase in patient and surgical volume, this does not suggest that HoLEP adoption failed to expand patient outreach. Furthermore, the lack of significant differences in prostate size between in-county and out-of-county, as well as in-state and out-of-state referrals, points to a broader regional under-treatment of larger prostate glands and a lack of HoLEP-trained practitioners in surrounding areas.

While barriers to the adoption of HoLEP still exist, there are demonstrated methods to decrease the effect of these barriers. With structured mentorship and HoLEP simulation, the previously described learning curve range of 50–60 cases can be shortened significantly, allowing earlier clinical independence.^12^,^19^ Moreover, HoLEP training in residency and fellowship will likely become more ubiquitous as academic urologists include HoLEP in their practice. Currently, 31% of residents report exposure to HoLEP during post-graduate training.^20^ In addition, the initial cost of specialized equipment and poor reimbursement rates have been identified as barriers to wider adoption. Our study provides evidence that these low rates can be overcome by increased surgical volume and low complication and reoperation rates. Moreover, one review detailed the successful use of reusable morcellators and ubiquitous low-power holmium lasers, significantly reducing cost.^14^ Notably, although some studies suggest reimbursement structures have traditionally disincentivized complex BPH care, our findings suggest that increased case volume may compensate for such financial incentives. Broader utilization of the procedure can also help drive economic forces to reduce equipment costs further. Finally, the reduced hospital stay length and catheterization times with HoLEP, compared to TURP, almost certainly translate to cost savings and efficiency.^21^ By highlighting the changes associated with the adoption of HoLEP, such as increased surgical volume and a rise in high-volume prostate BPH referrals, our findings provide insight into the types of surgical practices that may benefit from incorporating HoLEP. These insights can also guide training programs to promote a broader adoption of HoLEP.

This retrospective analysis is unique in its direct comparison of a surgeon’s practice before and after implementing HoLEP. However, this study has several limitations. Foremost, the data mirror only the experience of a single self-taught surgeon with academic support. Variation in referral patterns and a lack of infrastructure and support needed for HoLEP adoption may result in differing changes in non-academic and community settings. Furthermore, community practices may be less inclined to undertake the learning curve necessary to develop expertise in HoLEP. However, as HoLEP training becomes more prevalent, HoLEP-trained urologists will likely continue to enter community practices. Further research will be warranted to identify similar post-adoption changes in community urologists in a non-tertiary care setting with differing referral patterns.

## 5. Conclusion

Analysis of a general urologist’s practice revealed a significant increase in both clinical and surgical volume after the adoption of HoLEP, largely driven by BPH referrals. While the demographics, including age and prostate size, were different after HoLEP adoption, the surgeon’s catchment area remained comparable after HoLEP adoption, with a greater number of individuals being referred from within the same county as the treatment center. These findings support the need for HoLEP implementation among urologists, especially for those looking to increase surgical volume and treat individuals with larger prostates within their current patient population.

## Data Availability

The data sets generated during and/or analyzed during the current study are not publicly available but are available from the corresponding author on reasonable request.
